# Drug therapy: Sildenafil for post-operative pulmonary hypertension and Eisenmenger syndrome - A brief review of literature and survey of expert opinion

**DOI:** 10.4103/0974-2069.41063

**Published:** 2008

**Authors:** Balu Vaidyanathan

**Affiliations:** Department of Pediatric Cardiology, Amrita Institute of Medical Sciences and Research Center, Kochi, India

## INTRODUCTION

The pharmacological agent Sildenafil belongs to a class of drugs called phosphodiesterase (PDE) inhibitors. Phosphodiesterases are enzymes which catalyze the hydrolysis of cyclic nucleoside monophosphates, viz. 3'5'-cAMP (cAMP) and 3'5'-cGMP (cGMP). To date, at least 11 isoforms of PDE have been discovered.[1] The differential distribution of PDE isoforms in various tissues as well as the selectivity of pharmacological agents is the basis for the potential tissue-specific effects of PDE inhibitors.

## MECHANISM OF ACTION

Sildenafil and the related compounds Tadalafil and Vardenafil, are selective inhibitors of the cGMP-hydrolyzing isoform 5.[2] PDE 5 isoform is found in maximal concentrations in the corpus cavernosum, vascular smooth muscle, skeletal muscle and in platelets. Sildenafil increases the levels of cGMP leading to activation of a cGMP dependent protein kinase, which leads to phosphorylation of calcium ion channels with the final consequence of a reduced cytosolic calcium concentration. These drugs can have potential effects on the other PDE isoforms (eg: isoform 6 in retina and isoform 3 on platelets) which can explain some of the side-effects of these compounds.[1]

## CLINICAL EFFECTS

Phosphodiesterase 5 inhibitors can have a range of effects due to a very wide distribution of the isoform in various body tissues. Sildenafil is the drug which has been most often studied in various clinical trials. The clinically relevant effects have been noticed on the pulmonary vasculature and the smooth muscle of the corpus cavernosum (in patients with erectile dysfunction). Several studies have documented a relaxant effect on the pulmonary vascular smooth muscle with lowering of pulmonary artery pressure and pulmonary vascular resistance in patients with various forms of pulmonary hypertension.[3–5] These drugs have also been shown to have effects on systemic blood pressure and systemic vascular resistance, coronary blood flow and myocardial contractility.[2,6]

## SIDE EFFECTS

The most commonly reported side-effects of sildenafil can be attributed to vasodilation, such as flushing, nasal congestion, headache, dizziness and hypotension. Relaxation of the lower esophageal sphincter causes dyspepsia and reflux related symptoms.[6] In combination with other nitric oxide donors, Sildenafil can produce potentially life-threatening hypotension.[6] Visual abnormalities such as blurred vision or increased light perception might be related to partial inhibition of PDE 6 in the cones and rods of the retina.[6] There have been reports of tonic-clonic seizures, transient ischemic attacks, strokes and transient global amnesia after sildenafil.[7]

Sildenafil potentiates inhibitory effects of nitric oxide donors on ADP dependent platelet aggregation.[6,8] Case reports of intracerebral hemorrhage, gastric variceal bleeding, epistaxis and hemorrhoidal bleeding associated with sildenafil treatment suggest that the effect of PDE 5 inhibition in platelets might not be negligible. There is a potential interaction between Sildenafil and other antiplatelets drugs like clopidogrel and dipyridamole.

## CLINICAL TRIALS

The effects of Sildenafil and related compounds on pulmonary vasculature has been the focus of several recent clinical trials. Most of the studies are conducted in patients with primary pulmonary hypertension.[9–10] The findings of the major clinical trials are summarized below:

### 1. Primary pulmonary hypertension:

Galie *et al*, reported a favorable effect on six-minute walk test, mean pulmonary artery pressure and WHO functional class in adult patients treated with Sildenafil.[9] The incidence of clinical worsening did not differ between patients treated with sildenafil and placebo and the study was not empowered to assess mortality. In another randomized, placebo-controlled, double blind crossover trial, Sastry *et al*,[10] reported significant improvement in exercise tolerance, cardiac index and quality of life in patients treated with sildenafil. Importantly, one patient died and another had syncope during the placebo phase, highlighting the potential risks in sudden withdrawal of the drug. Sildenafil was relatively well tolerated in both the studies.

### 2. Eisenmenger syndrome:

This is characterized by elevated pulmonary vascular resistance resulting in a bidirectional or right-to-left shunt though a systemic-to-pulmonary circulation connection. The pathological changes in the pulmonary vascular bed are similar to those in patients with primary pulmonary hypertension.[11] In contrast to patients with primary pulmonary hypertension, the concern regarding the use of Sildenafil and related compounds in patients with Eisenmenger syndrome includes the potential risk of worsening hypoxia due to reduction in the systemic vascular resistance (SVR) and increase in right-to-left shunt.[12] In a preliminary observational study, Mukhopadhyay *et al*, demonstrated a significant decrease in pulmonary vascular resistance with improvement in systemic saturation, six-minute walk distance and WHO functional class in a group of 16 symptomatic patients with Eisenmenger syndrome treated with Tadafil.[13] None of the patients had a fall in systemic arterial pressure, worsening of oxygen saturation or adverse reactions to the drug.

### 3. Post-operative pulmonary hypertension

There are very few randomized trials on the use of Sildenafil and related drugs in post-operative pulmonary hypertension after surgery for congenital heart disease.[14–18] Stocker *et al*, in a prospective randomized trial of 16 ventilated infants early after closure of ventricular septal defect and atrioventricular septal defect reported significant reduction in pulmonary vascular resistance index when Sildenafil was used in conjunction with inhaled nitric oxide.[14] Sildenafil reduced the SVR and systemic blood pressure and worsened arterial oxygenation and alveolar-arterial gradient. The major limitation of this study was the small sample size and the fact that none of the patients had clinically significant pulmonary hypertension at the time of the study. Sculze-Neick *et al*, in a prospective non-randomized trial demonstrated that intravenous Sildenafil is more effective in reducing the PVR than nitric oxide.[5] Atz *et al*, showed that Sildenafil augments the effect of inhaled nitric oxide in postoperative pulmonary hypertensive crisis and suggested a role for Sildenafil during the phase of weaning from nitric oxide.[17] A recent meta-analysis concluded that oral Sildenafil maybe useful in reducing pulmonary vascular resistance and could be considered for treatment of postoperative pulmonary hypertension after pediatric heart surgery.[19] However, they cautioned that evidence from a large, multi-center, randomized controlled trial is needed to validate the safety and efficacy of Sildenafil for use in this setting.

Survey on Clinical Use of Sildenafil for Post Operative Pulmonary Hypertension and Eisenmenger Syndrome:

Based on the published literature in adults with primary pulmonary hypertension, there is widespread use of Sildenafil in pediatric patients with pulmonary hypertension (both idiopathic and post-operative pulmonary hypertension) and in patients with Eisenmenger syndrome. This scenario is particularly true in developing countries with limited access to drugs like Bosentan and prostaglandin analogues. Various issues related to the use of Sildenafil such as its efficacy, duration of treatment and safety have not been fully resolved

We therefore sought the opinion of some of the leading experts in the field of pulmonary hypertension in CHD with regard to the safety and efficacy of Sildenafil in two principal settings in congenital heart disease - postoperative pulmonary hypertension and Eisenmenger syndrome. The experts were invited to join an email survey on the use of Sildenafil in these two settings. Through a pre-designed questionnaire, a set of questions were put forward and the opinion of the experts regarding each of the issues was solicited.

The e-mail survey covered the following issues:

1. Postoperative pulmonary hypertension:
  - Should Sildenafil be administered for early postoperative pulmonary hypertension?
  - If so, what would be the indications and clinical conditions?
  - What is the dosage schedule that you use?
  - How long do you administer it? What are your criteria for stopping the medication?
  - What adverse effects have you encountered?
2. Eisenmenger syndrome:
  - Do you administer Sildenafil in patients with Eisenmenger syndrome? Choose one of the following:
    - Never
    - Routinely
    - In specific circumstances only
  - If your answer is iii, what are the specific circumstances?
  - Are you concerned about the potential for increased flow due to falling PVR resulting in increasing shear stress on the pulmonary vasculature?
  - What is the recommended duration of therapy?
  - How do you follow-up these patients?
  - Have you found Sildenafil effective in these patients?

Below is a summary of the expert opinions collected from this email survey:

### A: Postoperative pulmonary hypertension:

#### Question: Should Sildenafil be administered for early post-operative PAH? If so, what would be the indications and clinical conditions?

Most of the experts agreed that there was a role for Sildenafil in the management of postoperative pulmonary hypertension. The principal settings where Sildenafil was potentially useful included:

1. Older patients (> 6 months) with large L-R shunts (large VSD, AVSD, AP window, Truncus)
2. Repair of obstructed TAPVC
3. Borderline cases of Bidirectional Glenn shunt or Fontan operation with elevated PA pressure.
4. Weaning from nitric oxide

However, two experts opined that there was insufficient data at present to support the use of Sildenafil in postoperative pulmonary artery hypertension. One of them strongly felt that the drug should not be administered pending a carefully conducted double blind placebo controlled randomized study.

#### Question: Dosage schedule

The response to this question was variable. The dose range recommended varied from 0.5 - 5 mg/kg/day. The frequency of administration recommended was six-eight hourly. Most experts recommended starting the drug in lower dose and then increasing the dose to the maximum tolerated dose.

It is believed that most of the receptors get saturated at a dose of 0.3 mg/kg and though higher doses are well tolerated, there may not be any definite advantage over the standard dose.

#### Question: Duration of therapy and criteria for stopping Sildenafil.

If used in an acute setting (post-op pulmonary hypertensive crisis which settles down in a few days), it was felt that Sildenafil can be stopped after 2-3 weeks. However, if there was persistent pulmonary hypertension at the time of discharge, the drug should be continued for at least three months. Occasional older patients with persistent pulmonary hypertension after repair of large shunts may require longer duration of therapy. One expert opined that there was no rationale for continuing Sildenafil if the pulmonary hypertension was mild or moderate in an otherwise asymptomatic patient. Two-dimensional echocardiography was recommended by most for follow-up assessment of these patients to assess pulmonary hypertension.

#### Question: Adverse effects encountered

Most respondents felt that the drug was well tolerated in their personal experience. Occasional cases of systemic hypotension and headache were observed with higher doses. One expert was wary of the use of Sildenafil in patients with sepsis in view of its property of augmenting the nitric oxide pathway.

### B: Eisenmenger syndrome

#### Question: When will you administer Sildenafil in patients with Eisenmenger syndrome?

Most of the survey participants agreed that Sildenafil may be administered in a symptomatic patient with Eisenmenger syndrome. The criteria for use included severe cyanosis (oxygen saturation < 75%), symptoms of hyperviscosity (Hb > 20 gm/dl) and right ventricular dysfunction.

#### Question: Are you concerned about the potential for increase in pulmonary blood flow after use of Sildenafil?

Most experts were not worried about this possibility. The explanation was that the PVR was fixed and irreversible in all these patients and hence even a slight increase in pulmonary blood flow would have only minimal effect on the already established changes in the pulmonary vasculature.

#### Question: What is the recommended duration of therapy?

The majority of participants agreed that the therapy should be continued indefinitely and perhaps lifelong. The possibility of sudden worsening of symptoms after stopping Sildenafil is known.[10]

#### Question: How do you follow-up these patients?

Most of them followed-up their patients 3-6 monthly. Parameters recommended for objective evaluation of treatment efficacy included oxygen saturation at baseline and after physical activity, exercise tolerance (six-minute walk test), echocardiographic assessment of pulmonary artery pressure, time to clinical worsening and hospitalization rates. One of the experts recommended the number of hemodilution sessions needed to control hyperviscosity-related symptoms as an index of efficacy of treatment as practiced in his unit.

#### Question: Have you found Sildenafil effective in these patients?

According to some respondents, Sildenafil was effective in as many as 50% patients with improvement in functional capacity (six-minute walk test) and oxygen saturations after its use.

## SUMMARY

In conclusion, the use of Sildenafil in the treatment of postoperative pulmonary hypertension and Eisenmenger syndrome is based on anecdotal reports and small observational studies. Till date, no randomized control trials have been conducted with Sildenafil in either of the settings. Though expert opinion in this survey favored the use of Sildenafil in selected patients, one should be extremely wary of supporting the widespread use of Sildenafil in unselected populations without proper case selection and monitoring. To quote one of the experts, Dr Gil Wernovsky, "This type of investigation/new therapy should only be done in the context of a well designed, placebo controlled, blinded randomized clinical trial. It is time for pediatric cardiology to enter the 21st century medicine and not just throw new medicines at our sickest patients".
